# Leukemia Types and Subtypes Analysis: Epidemiological Age-Standardized Exploration in the Mexican Bajio Region

**DOI:** 10.3390/medicina60050731

**Published:** 2024-04-28

**Authors:** Pablo Romero-Morelos, Ana Lilia González-Yebra, Luis Jonathan Bueno-Rosario, Beatriz González-Yebra

**Affiliations:** 1 Departamento de Investigación, Universidad Estatal del Valle de Ecatepec, Ecatepec 55210, Estado de México, Mexico; 2 Departamento de Ciencias Aplicadas al Trabajo, División Ciencias de la Salud, Universidad de Guanajuato, Campus León, León 37670, Guanajuato, Mexico; 3 Unidad de Investigación, Hospital Regional de Alta Especialidad del Bajío, Servicios de Salud del Instituto Mexicano del Seguro Social para el Bienestar (IMSS-BIENESTAR), León 37544, Guanajuato, Mexico; 4 Departamento de Medicina y Nutrición, División Ciencias de la Salud, Universidad de Guanajuato, Campus León, León 37670, Guanajuato, Mexico

**Keywords:** leukemia, leukemia subtypes, Mexico, prevalence, age-standardized incidence rate

## Abstract

*Background and Objectives*: Leukemia, characterized by abnormal leukocyte production, exhibits clonal origin from somatic mutations. Globally, it ranked 15th in cancer incidence in 2020, with higher prevalence in developing countries. In Mexico, it was the ninth most frequent cancer. Regional registries are vital for understanding its epidemiology. This study aims to analyze the prevalence and age-standardized incidence rates of leukemias in a tertiary care hospital in the Mexican Bajio region. *Materials and Methods*: Leukemia cases from 2008–2018 were analyzed, and 535 medical records were included in this study. The prevalence, distribution, and age-specific incidence rate of different types and subtypes of leukemia were determined according to sex and age groups. *Results*: Overall, 65.79% consisted of lymphocytic leukemia, 33.64% of myeloid leukemia, and 0.56% of monocytic leukemia. No significant sex-based differences were found, but age-specific patterns were observed. Leukemia distribution by age revealed significant associations. Lymphocytic leukemia dominated in the pediatric population, particularly acute lymphocytic leukemia, while myeloid leukemia shifted towards adulthood. Age-specific incidence patterns showed, first, that lymphocytic leukemia is the most common leukemia in pediatric ages, and second, there is a shift from acute lymphocytic leukemia dominance in pediatric ages to myeloid leukemia incidence in late adulthood, emphasizing nuanced epidemiological dynamics. *Conclusions*: Acute leukemia cases occurred with high prevalence in our study population, with a high incidence in pediatric and adulthood populations, especially for acute lymphocytic leukemia, showing a (<18 years) 153.8 age-standardized incidence rate in the pediatric group, while in the adult population, the age-standardized rate was 59.84. In the age-specific analysis, we found that the childhood group (5–9 years) were the most affected by acute lymphocytic leukemia in the pediatric population, while in the adult population, the early-adulthood group (15–29 years) were the most affected age group. In contrast, chronic myeloid leukemia affected both adults and the pediatric populations, while chronic lymphocytic leukemia and monocytic leukemia were exclusive to adults. The study underscores the need for tailored diagnostic, treatment, and preventive strategies based on age, contributing valuable insights into the leukemia epidemiology of the Bajio region.

## 1. Introduction

Leukemia, a hematologic neoplasm characterized by the excessive production of immature or mature blood cells, encompasses various subgroups based on cell lineage and maturation status. At the molecular level, this neoplasm originates clonally, arising from somatic mutations in hematopoietic stem cells [1,2,3].

The epidemiology of leukemia varies geographically, with developing countries bearing the greatest burden. In 2020, leukemia ranked 15th globally in cancer incidence, with the 11th highest mortality rate related to cancer [4]. In Mexico, it ranked ninth in cancer incidence and seventh in cancer-related mortality [5,6,7].

Leukemia types and subtypes exhibit variability across regions, influenced by factors such as sex and age group [8]. Therefore, regional population-based registries are essential for understanding their prevalence, distribution, incidence, and mortality [9]. Notably, Mexico lacks a national system providing epidemiological data on these neoplasms [10,11,12]. Thus, this study aims to analyze the prevalence and age-standardized incidence rates of leukemias in a tertiary care hospital in the Mexican Bajio region.

## 2. Materials and Methods

All medical records of patients diagnosed with leukemia from 2008 to 2018 at the Bajio Regional High Specialty Hospital were analyzed. Leukemia was classified according to the International Classification of Diseases (ICD-10) into its different types: lymphocytic leukemia (LL), myeloid leukemia (ML), and monocytic leukemia (MoL), along with their respective subtypes: acute LL (ALL), chronic LL (CLL), acute ML (AML), chronic ML (CML), acute MoL (AMoL), and chronic MoL (CMoL). The confirmatory diagnosis was made through bone marrow examination and immunophenotyping for acute leukemias, bone marrow examination and karyotyping for chronic leukemias, and immunophenotyping for chronic lymphocytic leukemia.

Initially, for epidemiological purposes, an analysis was performed to examine the distribution of leukemia types and subtypes across different years, aiming to provide an overarching overview of the number of leukemia cases treated at the Bajio Regional High Specialty Hospital. The prevalence (n group/total N) and distribution of the different types and subtypes of leukemia were then analyzed by sex and age. The analyzed population was subsequently stratified into pediatric (≤18 years) and adult (>18 years) cohorts to assess the distribution of leukemia types and subtypes broadly. A finer-grained analysis was then conducted by partitioning both pediatric and adult groups into more specific age categories according to the National Institute of Geography and Statistics (Instituto Nacional de Estadística y Geografía—INEGI), namely, infancy (0–4 years), childhood (5–9 years), adolescence (10–14 years), early adulthood (15–29 years), adulthood (30–64 years), and late adulthood (>65 years). This approach aimed to enhance the precision of observations regarding any significant or insignificant variances in the distribution of different types and subtypes of leukemia.

Additionally, crude age incidence rate (cAIR) and age-standardized incidence rate (ASIR) per 100,000 inhabitants were calculated for both age groups. The age group population data were obtained from INEGI according to the 2022 census of the Mexican Bajio region.

Statistical analyses were conducted using the Shapiro–Wilk test for distribution analysis, the Mann–Whitney U test for age and sex analysis, and the Kruskal–Wallis test for analyzing age distribution across leukemia types or subtypes. The association between leukemia types and subtypes and sex, as well as age at diagnosis, was examined using contingency tables and analyzed using the chi-square test. A statistical significance of *p* < 0.05 and 95% confidence intervals were considered in the statistical analyses using GraphPad Prism v9.0.

This retrospective sub-study, derived from a main study (CI/HRAEB/014/2018) conducted at the Bajio Regional High Specialty Hospital, was approved by the research ethics committee of the hospital. As this sub-study was based on a retrospective review of medical records collected from patients upon admission to the hospital, informed consent was not required.

## 3. Results

### 3.1. Overall Prevalence of Leukemia Types and Subtypes by Year

A total of 535 leukemia cases were identified from 2008 to 2018, with 65.79% (n = 352) corresponding to LL, 33.64% (n = 180) to ML, and 0.56% (n = 3) to MoL. This indicates an upward trend in total leukemia cases over the 10-year period, with 2017 recording the highest number of cases at 14% (n = 75), followed by 2018 (11.9%) and 2016 (11.4%). Analysis of leukemia subtypes by year reveals that ALL (63.36%) had the highest prevalence during the study period, followed by AML (24.11%) and CML (9.53%) (Table 1).

### 3.2. Distribution and Prevalence of Leukemia Types and Subtypes by Sex and Age at Diagnosis

No significant association (*p* = 0.99) was observed between the different types of leukemia and sex, with 277 males and 258 females included in the analysis. In terms of distribution by sex, males comprised 65.7% (n = 182) of LL cases, 33.6% (n = 93) of ML cases, and 0.7% (n = 2) of MoL cases. On the other hand, females accounted for 65.9% (n = 170) of LL cases, 33.7% (n = 87) of ML cases, and 0.4% (n = 1) of MoL cases.

**Figure 1:**
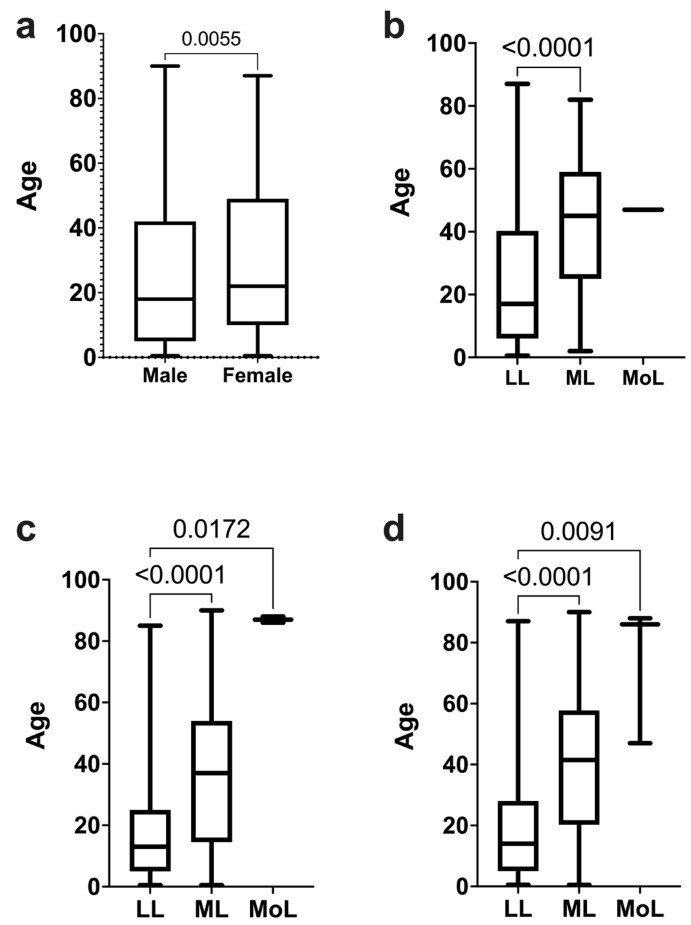
Age distribution in leukemia patients. (**a**) Comparative analysis between male and female sexes. (**b**) Comparative analysis of the diagnosis age for leukemia types (lymphoid, myeloid, or monocytic) in females. (**c**) Comparative analysis of the presentation age for leukemia types (lymphoid, myeloid, or monocytic) in males. (**d**) Analysis of leukemia types at different ages, including both sexes.

Analysis of the age at leukemia diagnosis by sex revealed that females were diagnosed at a median age of 22 years, whereas males were diagnosed at a median age of 18 years (Figure 1a).

Further analysis of leukemia type and age at diagnosis indicated that female LL and ML patients had median ages of 17 and 45 years, respectively, showing significant differences (p < 0.0001). However, the median age for female MoL patients was 47 years, though this difference was not statistically significant compared to other leukemic types (Figure 1b).

For male patients, LL was diagnosed at a significantly younger age, with a median of 13 years, compared to ML at 37 years (*p* < 0.0001). MoL was diagnosed at a median age of 37 years for males, with significant differences in age observed between LL and MoL (*p* = 0.0172) (Figure 1c).

When considering the age at diagnosis for both male and female patients, LL (median of 14 years) was found to manifest at a younger age compared to ML (median of 41.5 years) (*p* < 0.001) and MoL (median of 86 years) (*p* = 0.0091) (Figure 1d).

**Figure 2:**
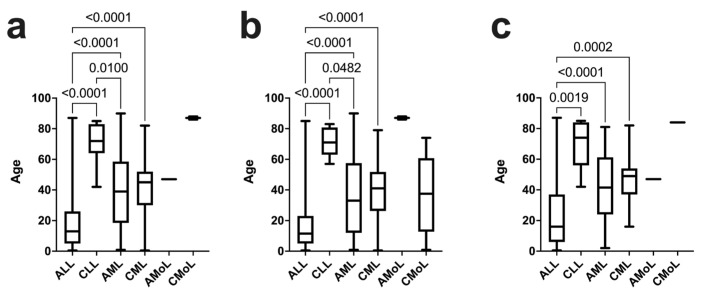
Age of presentation for different subtypes of leukemia. (**a**) Diagnosis age of leukemia subtypes in both sexes. (**b**) Age of presentation of leukemia subtypes in the male population. (**c**) Age of presentation of leukemia subtypes in the female population.

Analysis of the age at diagnosis of leukemia subtypes in both male and female patients revealed that ALL occurred at a younger age (median of 13 years) compared to the other leukemia subtypes (*p* < 0.01), except for MoL. CLL manifested at a more advanced age (median of 72 years) compared to ALL and AML (median of 40 years) (*p* < 0.01). CMoL was the subtype that occurred at the most advanced age (median of 87 years), although this finding was not statistically significant. AML exhibited a widespread distribution in both childhood and adulthood (Figure 2a).

Further analysis of leukemia subtypes by sex demonstrated that ALL occurred at a younger age (median of 11.5 years) in males compared to all other subtypes (*p* < 0.05), while CMoL and CLL occurred at an older age (median of 71 years and 87 years, respectively) and exclusively in adulthood. Notably, both AML and CML occurred across all ages (median of 33 years and 41 years, respectively) (Figure 2b).

Regarding female patients, ALL manifested at a younger age (median of 16 years) (*p* < 0.001) compared to the other leukemia subtypes, except for AMoL (median of 47 years) (*p* = 0.6628). CLL appeared at the most advanced age (median of 74 years) and exclusively in patients above 40 years, while AML and CML occurred in patients aged 2 to 79 years (median of 41.5 years and 49 years, respectively) (Figure 2c).

### 3.3. Distribution, Prevalence and Age Incidence Rates of Leukemia Types and Subtypes in the Pediatric (≤18 Years) and Adult (>18 Years) Groups

MoL cases were excluded from this analysis due to their limited occurrence (only three instances). An association was found between the pediatric population and LL (*p* < 0.0001) with a relative risk (RR) of 1.716 (95% CI 1.513–1.964).

Regarding the distribution of leukemia types, LL accounted for 84.9% (n = 202) of cases in the pediatric population and 50.5% (n = 150) in adults, while ML occurred in 15.1% (n = 36) of the pediatric population and 48.5% (n = 144) of adults (Table 2).

Analysis of leukemia subtypes revealed a significant association (*p* < 0.0001) between the pediatric population and ALL, with an RR of 1.66 (95% CI 1.474–1.891). In the pediatric population, ALL constituted 84.9% (n = 202) of cases, while adults accounted for 46.1% (n = 137) of ALL cases. Additionally, the pediatric population had 0% of CLL cases, 12.6% (n = 30) of AML cases, and 2.5% (n = 6) of CML cases, whereas adults had 4.4% (n = 13) of CLL cases, 33.3% (n = 99) of AML cases, 15.2% (n = 45) of CML cases, 0.7% (n = 2) of CMoL cases, and 0.3% (n = 1) of AMoL cases (Table 2).

According to INEGI data, the total population in the Bajio region was 18,293,446 inhabitants, with 6,183,185 in the pediatric group and 12,110,261 in the adult group. In the pediatric group, the crude age incidence rate (cAIR) was 3.27 for LL and 0.58 for ML. The age-standardized incidence rate (ASIR) per 100,000 inhabitants was 153.87 for LL and 27.42 for ML.

In the adult group, the cAIR was 1.24 for LL, 1.19 for ML, and 0.02 for MoL. The ASIRs per 100,000 inhabitants were 65.52 for LL, 62.9 for ML, and 1.31 for MoL. LL and ML were the most common leukemias in this age group.

Regarding leukemia subtypes in the pediatric group, the cAIR was 3.27 for ALL, 0.49 for AML, and 0.1 for CML. The ASIRs in this group were 153.87 for ALL, 22.85 for AML, and 4.57 for CML.

In the adult group, the cAIR was 1.13 for ALL, 0.11 for CLL, 0.82 for AML, 0.37 for CML, 0.02 for CMoL, and 0.01 for AmoL. The ASIRs were 59.84 for ALL, 5.68 for CLL, 43.25 for AML, 19.66 for CML, 0.87 for CMoL, and 0.44 for AMoL (Table 2).

### 3.4. Distribution, Prevalence, and Incidence Rates of Leukemia Types and Subtypes by Age Group Classification

Based on the associations previously identified in the pediatric population (≤18 years) between LL and ALL, along with the high age-standardized incidence rates (ASIRs) of these leukemia types and subtypes in both the pediatric and adult groups, an analysis was conducted by age group according to the INEGI classification to determine the distribution, prevalence, and incidence rates of leukemia types and subtypes in specific age groups.

**Figure 3:**
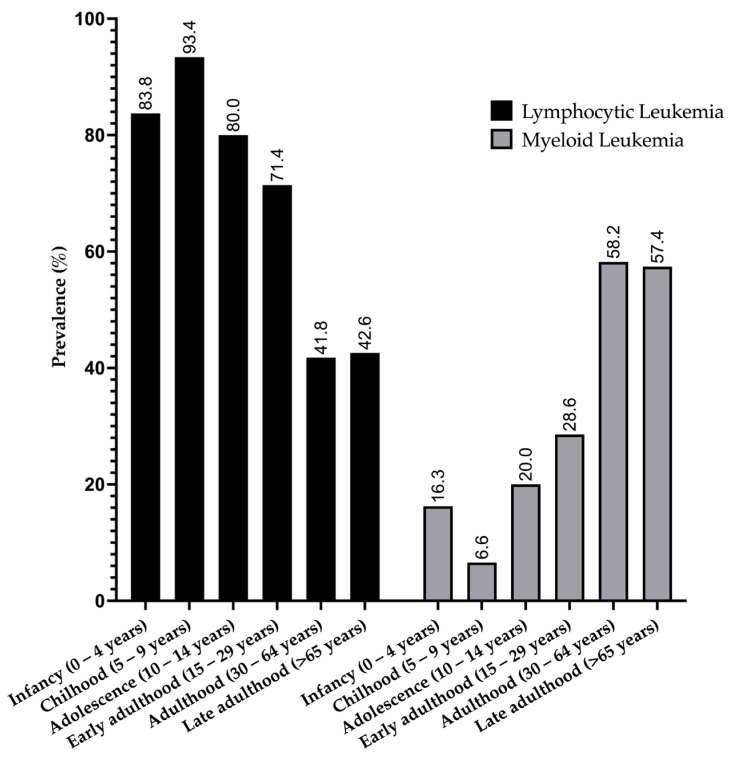
Prevalence of leukemia types in different age groups.

A peak in LL cases was observed in the childhood group, accounting for 93.4% of cases, with a subsequent decline in prevalence within older age groups. The lowest prevalence of LL (41.8%) was found in the adulthood group. Conversely, for ML cases, the age group with the lowest prevalence was childhood (6.6%), with an increase observed in older age groups. The highest prevalence of ML (58.2%) was identified in the adulthood group (Figure 3).

**Figure 4:**
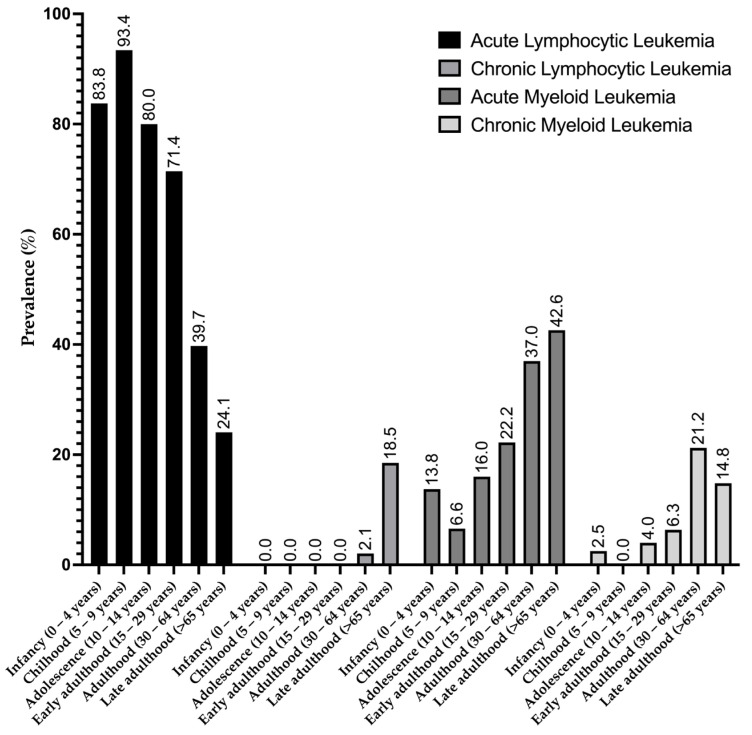
Prevalence of leukemia subtypes in different age groups.

Analysis of leukemia subtypes revealed that ALL exhibited the highest prevalence in the childhood group (93.4%), with a gradual decrease in older age groups, and the lowest prevalence was observed in the late-adulthood group (24.1%). Conversely, CLL occurred only from adulthood onwards (2.1%), reaching its peak in the late-adulthood group (18.5%). AML had its lowest prevalence in the childhood group (6.6%), with an increase observed in subsequent age groups and a peak in the late-adulthood group (42.6%). CML was most prevalent in the adulthood group (21.2%), followed by late adulthood (Figure 4).

The total inhabitants per age groups were as follows: 2,122,040 for infants, 2,067,159 for childhood, 1,993,986 for adolescents, 4,061,145 for early adults, 7,079,564 for adults, and 9,695,553 for late adults.

In the infant group, the most common leukemia type was LL (ASIR = 36.63), and the most common subtype was ALL (ASIR = 36.63). Similarly, in childhood, LL was the most common leukemia type (ASIR = 38.81), and ALL was the most common leukemia subtype (ASIR = 38.81). In adolescence, LL and ALL were the most common leukemia type and subtype (ASIR = 22.41), followed by ML (ASIR = 5.47) for leukemia types and AML (ASIR = 4.37) for leukemia subtypes (Table 3).

On the other hand, in early adulthood, the most common leukemia type and subtype were LL and ALL (ASIR = 48.65), followed by ML (ASIR = 19.68) and AML (ASIR = 15.31). In adulthood, the most common leukemia type was ML (ASIR = 46.46), followed by LL (ASIR = 33.35). For leukemia subtypes, ALL was the most common (ASIR = 31.37), followed by AML (ASIR = 29.52). In late adulthood, the most common leukemia type was ML (ASIR = 16.95), and the most common leukemia subtype was AML (ASIR = 12.57).

These results indicate an inversion in the incidence of leukemia types in adult groups, with LL being the most common type only in early adulthood, later (in adulthood and late adulthood) being replaced by ML as the most common type (Table 4).

## 4. Discussion

Our study provides a comprehensive analysis of leukemia epidemiology in the Bajio region over the past decade, offering insights into the incidence, prevalence, distribution, and age-specific patterns of different leukemia types and subtypes.

For the prevalence of leukemia types and subtypes by year, we analyzed leukemia cases reported at the Bajio Regional High Specialty Hospital from 2008 to 2018 and found an increase in the number of cases of about 7.5% per year, which is higher than the 0.6% yearly increase reported globally [13,14]. This could be due to a gradual increase in patient recruitment at our hospital because it was recently established in 2008 as a high-specialty hospital in the Bajio region, although it is not an oncology center.

Interestingly, 2017 emerged as the peak year for leukemia cases, accounting for 14% of all diagnoses. The rise in prevalence during this period might be attributed to various factors, such as increased awareness, improved diagnostic capabilities, or environmental factors [15,16]. This temporal trend should be closely monitored for continued surveillance and to identify potential contributing factors.

Our analysis of leukemia cases by sex revealed no significant association, with both males and females exhibiting similar prevalence rates across LL, ML, and MoL subtypes. However, age-specific patterns demonstrated distinct characteristics. Females were diagnosed at an older age overall, with variations observed among leukemia subtypes. The finding that male LL patients were diagnosed at a significantly earlier age than their female counterparts suggests potential gender-specific differences in disease onset [17,18].

Regarding the distribution and prevalence of different types of leukemia according to sex, no significant differences were found, although global statistics indicate that leukemia is more frequent and deadly in men than in women [7].

As for the overall prevalence of leukemia types and subtypes, these are consistent with those reported in other studies [6,7,9], with LL cases ranking first and ML second. Regarding leukemia subtypes, ALL ranked first and AML second, followed by CML.

We found ALL was the most frequent leukemia subtype in pediatric groups, even up to the age of 29 years, which is consistent with global reports that the pediatric population has the highest prevalence of ALL cases. It is important to note that the Hispanic pediatric population has shown an interesting susceptibility to ALL, including in the United States, where it occurs more frequently in infants of Hispanic origin, while it is the most frequent type of leukemia in Mexico at the age of 1–4 years [19]; nevertheless, in our study, we found a peak in ALL in the 5–9-years age group. In this sense, bimodal behavior has been reported for ALL, that is, it can be frequently found in two age groups, with a first peak at the age of 0–4 years and a second peak at the age of 75 years, predominantly in the male population; however, these peaks were not observed in our study [14].

Our study delves into leukemia subtypes, revealing notable differences in age at diagnosis. ALL consistently occurred at a younger age compared to other subtypes, emphasizing the importance of subtype-specific considerations in diagnosis and treatment planning. The occurrence of CLL and CMoL exclusively in adulthood and at an advanced age suggests distinct pathophysiological mechanisms and warrants further investigation into the etiology of these subtypes in older populations.

As for the distribution and prevalence of leukemia types and subtypes in adult and pediatric populations or by age groups, studies indicate that AML is frequent in adults and has a poor prognosis, while CML is also frequent in this age group but has a better prognosis. These leukemia subtypes have also been found in the pediatric population but with a lower prevalence, which is consistent with the findings of our study [14,20,21].

The literature indicates that CML occurs with a prevalence of 15% in the United States, mainly affecting the adult population aged 65–74 years, which is similar to what was found in our study (14.3%) [13,14].

The analysis of leukemia distribution in pediatric vs. adult populations revealed significant associations. LL had a higher prevalence in the pediatric population, particularly ALL, highlighting the distinct landscape of leukemia in childhood. Conversely, ML demonstrated a shift towards adulthood, aligning with previous literature that outlines the varied prevalence of leukemia subtypes across different age groups [13,20].

In this sense, our study delved into age-specific (infancy, childhood, adolescence, early adulthood, adulthood, and late adulthood) incidence patterns, revealing intriguing nuances across distinct age groups. In the pediatric population, ALL dominated in infancy and childhood, gradually giving way to a more balanced incidence of ALL and ML in adolescence. Meanwhile, early adults faced a higher incidence of ALL and LL, reflecting a shift in leukemia patterns during this transitional age.

In contrast, adults witnessed a considerable incidence of ML, emphasizing its prevalence in this demographic. Late adults, however, experienced a distinct inversion, with ML maintaining its lead, accompanied by an increased incidence of AML. This age-specific inversion underscores the dynamic nature of leukemia epidemiology, suggesting that the factors contributing to leukemia development may differ between pediatric and adult age groups, presenting a compelling area for further research.

According to incidence rates of leukemia types and subtypes, there are some studies from the country’s leading health institutions that have analyzed age-adjusted incidence rates in leukemia, showing a consensus that ALL is the most common subtype in individuals under 18 years old, with raw incidence rates close to 40 cases per million inhabitants, as we found when analyzing cases in the Mexican Bajio region [22,23].

To date, no study has explored incidence rates for both types and subtypes of leukemia in the country across different age groups. Peaks in incidence at specific ages have been observed, such as in the case of ALL, with higher incidences in childhood, adolescence, early adults, and adulthood. This may be influenced by the population distribution in these age groups, as in the case of the Mexican Bajio, where these age groups represent most of the population. Additionally, it has been suggested that this type of leukemia may be linked to the presence of environmental factors, such as exposure to volatile organic compounds, which are common in the region due to the prevalent leather industry in the Bajio; however, more studies should be performed in this regard.

## 5. Conclusions

Understanding the epidemiological landscape of leukemia in the Bajio region is crucial for informing clinical practice, public health interventions, and future research endeavors. Our study reveals that acute leukemia cases occurred with high prevalence in our population, exhibiting a significant incidence in both pediatric and adult populations, particularly for acute lymphocytic leukemia (ALL). In the pediatric population, the ASIR for ALL was 153.8 cases per 100,000 inhabitants, whereas in the adult population, the age-standardized incidence rate was 59.84. Through age-specific analysis, we determined that the childhood group (5–9 years) was most affected by ALL in the pediatric population, while in the adult population, the early-adulthood group (15–29 years) exhibited the highest incidence. Chronic myeloid leukemia (CML) affected both adults and pediatric populations, while chronic lymphocytic leukemia (CLL) and monocytic leukemia were exclusive to adults. The observed age-specific patterns and gender differences underscore the need for tailored approaches in diagnostics, treatment, and prevention strategies. Our comprehensive analysis contributes valuable insights into the nuanced epidemiology of leukemia in the Bajio region. The identified temporal trends, age-specific patterns, and subtype-specific variations provide a solid foundation for future research aimed at unraveling the multifaceted factors contributing to leukemia development in this population. Further investigation into the etiology, risk factors, and molecular mechanisms underlying leukemia subtypes in distinct age groups will enhance our understanding and inform targeted interventions to improve patient outcomes and reduce the burden of leukemia in the Bajio region. However, it is important to consider that one of the main limitations of this study is that the results obtained are from a single center, including only the cases recorded at the Regional High Specialty Hospital of Bajio. Therefore, the results obtained may be due to isolated behaviors and should be explored and validated in a larger, multicenter population of the region.

## Figures and Tables

**Table 1:** Prevalence of leukemia subtype cases per year.

Year			Lymphocytic				Myeloid				Monocytic			
	Total Sample		Acute		Chronic		Acute		Chronic		Acute		Chronic	
	n	%	n	%	n	%	n	%	n	%	n	%	n	%
2008	20	3.74	9	1.68	0	0.00	7	1.31	3	0.56	0	0.00	1	0.19
2009	42	7.85	25	4.67	1	0.19	13	2.43	3	0.56	0	0.00	0	0.00
2010	47	8.79	33	6.17	0	0.00	9	1.68	4	0.75	1	0.19	0	0.00
2011	51	9.53	37	6.92	1	0.19	9	1.68	3	0.56	0	0.00	1	0.19
2012	59	11.03	43	8.04	1	0.19	9	1.68	6	1.12	0	0.00	0	0.00
2013	38	7.10	22	4.11	3	0.56	7	1.31	6	1.12	0	0.00	0	0.00
2014	38	7.10	27	5.05	0	0.00	7	1.31	4	0.75	0	0.00	0	0.00
2015	40	7.48	23	4.30	0	0.00	13	2.43	4	0.75	0	0.00	0	0.00
2016	61	11.40	33	6.17	3	0.56	19	3.55	6	1.12	0	0.00	0	0.00
2017	75	14.02	50	9.35	2	0.37	16	2.99	7	1.31	0	0.00	0	0.00
2018	64	11.96	37	6.92	2	0.37	20	3.74	5	0.93	0	0.00	0	0.00
Total	535	100	339	63.36	13	2.43	129	24.11	51	9.53	1	0.19	2	0.37

Data are expressed in absolute frequence and prevalence. Prevalence was determined by subtype and year, n: number of patients, (%): Prevalence.

**Table 2:** Leukemia incidence in pediatric and adult patients.

	<18 (N = 238)			≥18 (N = 297)			Total (N = 535)
Leukemia Type	n (%)	cAIR	ASIR	n (%)	cAIR	ASIR	
LL	202.0 (84.9%)	3.27	153.87	150.0 (50.5%)	1.24	65.52	352.0 (65.8%)
ML	36.0 (15.1%)	0.58	27.42	144.0 (48.5%)	1.19	62.90	180.0 (33.6%)
MoL	0.0 (0.0%)	0.00	0.00	3.0 (1.0%)	0.02	1.31	3.0 (0.6%)
Leukemia Subtype	n (%)	cAIR	ASIR	n (%)	cAIR	ASIR	
ALL	202.0 (84.9%)	3.27	153.87	137.0 (46.1%)	1.13	59.84	339.0 (63.4%)
CLL	0.0 (0.0%)	0.00	0.00	13.0 (4.4%)	0.11	5.68	13.0 (2.4%)
AML	30.0 (12.6%)	0.49	22.85	99.0 (33.3%)	0.82	43.25	129.0 (24.1%)
CML	6.0 (2.5%)	0.10	4.57	45.0 (15.2%)	0.37	19.66	51.0 (9.5%)
CMoL	0.0 (0.0%)	0.00	0.00	2.0 (0.7%)	0.02	0.87	2.0 (0.4%)
AMoL	0.0 (0.0%)	0.00	0.00	1.0 (0.3%)	0.01	0.44	1.0 (0.2%)

Pediatric patients ≤ 18; Adult patients > 18; cAIR = crude age incidence rate; ASIR = age-standardized incidence rate. Incidence rate per 100,000 inhabitants. LL = lymphocytic leukemia, ML = myeloid leukemia, MoL = monocytic leukemia ALL = acute lymphocytic leukemia, CLL = chronic lymphocytic leukemia, AML = acute myeloid leukemia, CML = chronic myeloid leukemia, AMoL = acute monocytic leukemia, CMoL = chronic monocytic leukemia.

**Table 3:** Leukemia incidence in pediatric age groups.

	Infancy (N = 80)			Childhood (N = 76)			Adolescence (N = 51)		
Type	n (%)	cAIR	ASIR	n (%)	cAIR	ASIR	n (%)	cAIR	ASIR
LL	67.0 (83.8%)	3.16	36.63	71.0 (93.4%)	3.43	38.81	41.0 (80.4%)	2.06	22.41
ML	13.0 (16.2%)	0.61	7.11	5.0 (6.6%)	0.24	2.73	10.0 (19.6%)	0.50	5.47
MoL	0.0 (0.0%)	0.00	0.00	0.0 (0.0%)	0.00	0.00	0.0 (0.0%)	0.00	0.00
Subtypes	n (%)	cAIR	ASIR	n (%)	cAIR	ASIR	n (%)	cAIR	ASIR
ALL	67.0 (83.8%)	3.16	36.63	71.0 (93.4%)	3.43	38.81	41.0 (80.4%)	2.06	22.41
CLL	0.0 (0.0%)	0.00	0.00	0.0 (0.0%)	0.00	0.00	0.0 (0.0%)	0.00	0.00
AML	11.0 (13.8%)	0.52	6.01	5.0 (6.6%)	0.24	2.73	8.0 (15.7%)	0.40	4.37
CML	2.0 (2.5%)	0.09	1.09	0.0 (0.0%)	0.00	0.00	2.0 (3.9%)	0.10	1.09
CMoL	0.0 (0.0%)	0.00	0.00	0.0 (0.0%)	0.00	0.00	0.0 (0.0%)	0.00	0.00
AMoL	0.0 (0.0%)	0.00	0.00	0.0 (0.0%)	0.00	0.00	0.0 (0.0%)	0.00	0.00

cAIR = crude age incidence rate; ASIR = age-standardized incidence rate; incidence rate per 100,000 inhabitants. LL = lymphocytic leukemia, ML = myeloid leukemia, MoL = monocytic leukemia ALL = acute lymphocytic leukemia, CLL = chronic lymphocytic leukemia, AML = acute myeloid leukemia, CML = chronic myeloid leukemia, AMoL = acute monocytic leukemia, CMoL = chronic monocytic leukemia.

**Table 4:** Leukemia incidence in adult age groups.

	Early Adulthood (N = 125)			Adulthood (N = 147)			Late Adulhood (N = 56)		
Type	n (%)	cAIR	ASIR	n (%)	cAIR	ASIR	n (%)	cAIR	ASIR
LL	89.0 (71.2%)	2.19	48.65	61.0 (41.5%)	0.86	33.35	23.0 (41.1%)	2.37	12.57
ML	36.0 (28.8%)	0.89	19.68	85.0 (57.8%)	1.20	46.46	31.0 (55.4%)	3.20	16.95
MoL	0.0 (0.0%)	0.00	0.00	1.0 (0.7%)	0.01	0.55	2.0 (3.6%)	0.21	1.09
Subtypes	n (%)	cAIR	ASIR	n (%)	cAIR	ASIR	n (%)	cAIR	ASIR
ALL	89.0 (71.2%)	2.19	48.65	58.0 (39.5%)	0.82	31.71	13.0 (23.2%)	1.34	7.11
CLL	0.0 (0.0%)	0.00	0.00	3.0 (2.0%)	0.04	1.64	10.0 (17.9%)	1.03	5.47
AML	28.0 (22.4%)	0.69	15.31	54.0 (36.7%)	0.76	29.52	23.0 (41.1%)	2.37	12.57
CML	8.0 (6.4%)	0.20	4.37	31.0 (21.1%)	0.44	16.95	8.0 (14.3%)	0.83	4.37
CMoL	0.0 (0.0%)	0.00	0.00	0.0 (0.0%)	0.00	0.00	2.0 (3.6%)	0.21	1.09
AMoL	0.0 (0.0%)	0.00	0.00	1.0 (0.7%)	0.01	0.55	0.0 (0.0%)	0.00	0.00

cAIR = crude age incidence rate; ASIR = age-standardized incidence rate. Incidence rate per 100,000 inhabitants. LL = lymphocytic leukemia, ML = myeloid leukemia, MoL = monocytic leukemia ALL = acute lymphocytic leukemia, CLL = chronic lymphocytic leukemia, AML = acute myeloid leukemia, CML = chronic myeloid leukemia, AMoL = acute monocytic leukemia, CMoL = chronic monocytic leukemia.

## Data Availability

The data that support the findings of this study are available on request from the corresponding author.
